# Low Humoral Immune Response and Ineffective Clearance of SARS-Cov-2 in a COVID-19 Patient With CLL During a 69-Day Follow-Up

**DOI:** 10.3389/fonc.2020.01272

**Published:** 2020-07-03

**Authors:** Xingnong Ye, Xiaofang Xiao, Bin Li, Weigang Zhu, Youjiang Li, Jianguo Wu, Xin Huang, Jingxia Jin, Dan Chen, Jie Jin, Jian Huang

**Affiliations:** ^1^Department of Hematology, The Fourth Affiliated Hospital of Zhejiang University School of Medicine, Zhejiang, China; ^2^Department of Hematology, The First Affiliated Hospital, Zhejiang University School of Medicine, Zhejiang, China; ^3^Key Laboratory of Hematologic Malignancies, Diagnosis and Treatment, Hangzhou, China; ^4^Department of Infectious Diseases, The Fourth Affiliated Hospital of Zhejiang University School of Medicine, Zhejiang, China; ^5^Department of Laboratory Medicine, The Fourth Affiliated Hospital of Zhejiang University School of Medicine, Zhejiang, China

**Keywords:** coronavirus disease-2019, chronic lymphocytic leukemia, humoral immunity, ineffective clearance, follow-up

## Abstract

**Background:** A recent outbreak of severe acute respiratory syndrome coronavirus 2 (SARS-Cov-2), which began in Wuhan, China, with a high level of human-to-human transmission has been reported. There are limited data available on Coronavirus Disease 2019 (COVID-19) patients with hematological malignancies with more than 60 days of follow-up. This study describes the clinical characteristics, including multiple recurrences of COVID-19, in a patient with chronic lymphocytic leukemia (CLL) during 69 days of follow-up.

**Case Presentation:** A 72-year-old female was admitted to hospital isolation after being infected with COVID-19 as part of a family cluster on January 30, 2020. Apart from SARS-Cov-2 virus infection, laboratory results revealed lymphocytosis of uncertain etiology and abnormal distribution of T lymphocytes. On blood smears, small blue lymphocytes with scant cytoplasm were observed, and the presence of high levels of circulating clonal B cells was also demonstrated by flow cytometry. The patient was diagnosed with COVID-19 and CLL. Among her family members, she had the highest viral loads and the fastest progression on lung injury and developed severe pneumonia. Serological results showed she had both 2019-nCoV-specific IgM and IgG antibodies; however, only IgG antibodies were detected in her husband's plasma.

**Results:** A combination regimen of antiviral therapy and high-dose intravenous immunoglobulin (IVIG) in the early stage seemed to be effective for treating CLL and SARS-Cov-2 infection. Because of the low humoral immune response, the CLL patient could not effectively clear the SARS-Cov-2 infection and suffered from recurrence twice during the 69-day follow-up.

**Conclusion:** In CLL, a neoplastic antigen-specific B-cell clone proliferates, and the progeny cells accumulate and outgrow other B cells, leading to immune deficiency. Considering the low humoral immune response and ineffective clearance of SARS-Cov-2 in CLL patients, the follow-up and home quarantine period should be extended. We need further studies to clarify suspending or continuing CLL therapy during COVID infection. For those patients who are prone to progression to severe disease, administering humoral immunity therapies can help to prevent disease progression and quickly meet the cure criteria.

## Background

Chronic lymphocytic leukemia (CLL) is a malignant hematological disorder characterized by the accumulation of single-clone B lymphocytes in peripheral blood, lymph nodes, and bone marrow (1). The diagnosis of CLL is based on the International Workshop on Chronic Lymphocytic Leukemia (IWCLL) criteria (2). Infectious complications due to immune dysfunction constitute the leading cause of mortality in patients with CLL. A neoplastic antigen-specific B-cell clone proliferates and outgrows other B cells, leading to humoral immune deficiency in CLL patients (3). Moreover, monoclonal cells affect the phenotype and function of a variety of innate and adaptive immune cells, including monocytes, T cells, and natural killer cells, leading to a tumor-supportive environment and reduced immune surveillance (4, 5). A study found that cellular immunotherapy impeded CLL, which may be related to acquired immune dysfunction that mainly manifests as abnormal expansion of T cells, failure to form synaptic T cells, and inhibition of T cell migration (6). Inhibition of immune surveillance increases the risk of viral infections (7).

During the recent outbreak of severe acute respiratory syndrome coronavirus 2 (SARS-Cov-2), which began in Wuhan, China, a high degree of human-to-human transmission via direct or indirect contact with large respiratory droplets and/or airborne transmission has been reported (8–10). In addition to fever, a variety of signs and symptoms, including cough, diarrhea, fatigue, and headache, may be presented, and infections can cause disease that ranges in severity from trivial colds and sore throats to serious laryngeal and tracheobronchial infections, bronchiolitis, and frank pneumonia (11). Studies have shown the general susceptibility of the population and have revealed the risk factors for the development of severe pneumonia, including neoplastic disorders, cardiovascular and cerebrovascular diseases, and advanced age (10, 12). There are limited data available on patients with coronavirus disease 2019 (COVID-19) and also hematological malignancies over more than 60 days of follow-up. In this study, we report the clinical characteristics, treatment, and multiple recurrence of COVID-19 in a patient with CLL during a 69-day follow-up.

## Case Presentation

**Figure 1:**
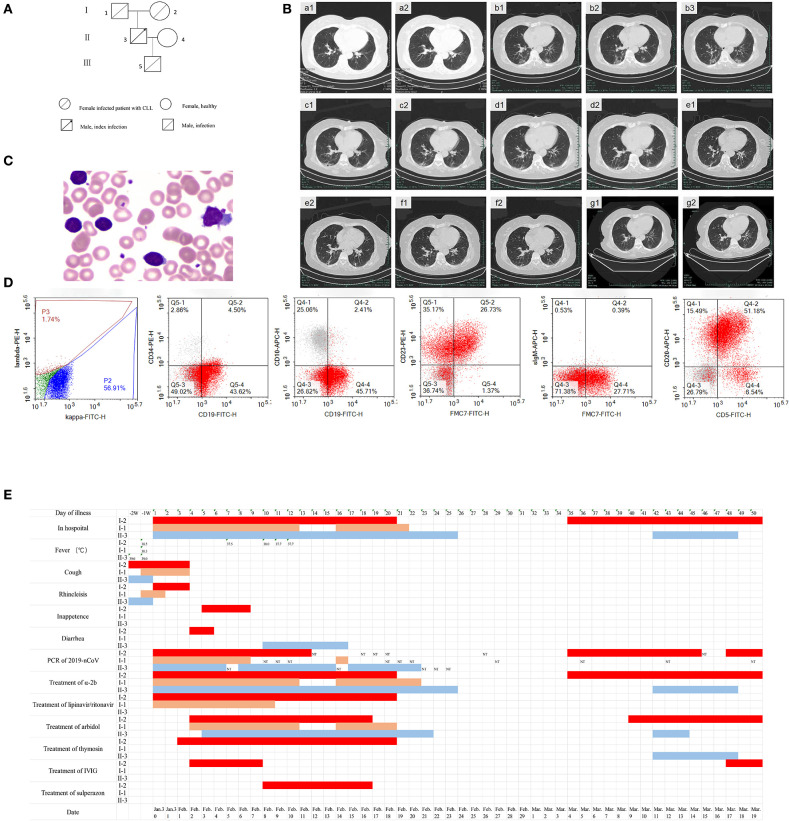
The clinical characteristics and treatment of a COVID-19 patient with chronic lymphocytic leukemia (CLL) (I-2) from a familial cluster during 69 days of follow-up. **(A)** Genealogical tree of patient I-2. **(B)** Chest CT of patient I-2 on day -7 (a1,a2), day 1 (b1,b2,b3), day 8 (c1,c2), day 18 (d1,d2), day 26 (e1,e2), day 35 (f1,f2), and day 47 (g1,g2). **(C)** Peripheral blood smear of patient I-2 (Wright's staining, oil-immersion lens, 1,000 ×). **(D)** Immunophenotyping of peripheral blood cells of patient I-2. **(E)** Time at home or in hospital, clinical symptoms, positive or negative novel coronavirus nucleic acid result, and the use of antiviral drugs and immune modulators for 69 days of follow-up of patients I-2, I-1, and II-3. a-2b: interferon α-2b, IVIG: intravenous immunoglobulin.

A 72-year-old female experienced the onset of COVID-19 after being infected as part of a household cluster. Figure 1A shows the genealogical family tree. II-3 was the index patient, and he was admitted to the hospital on January 30, 2020, with a consistent cough that had lasted for 10 days. A nasopharyngeal swab was positive for SARS-Cov-2. Nasopharyngeal swabs from three males (I-1, II-3, and III-5) and 1 female (I-2) in the household were also positive for SARS-Cov-2. On admission, I-2 had no complaints, with no cough, fever, or shortness of breath. Four months prior, a blood analysis at a local hospital showed that she had increased lymphocytes (34.22 × 10^9^/L, range, 1.1–3.2) and normal counts of hemoglobin and platelets during a regular medical examination. On January 23, she had a moderate fever with a mild cough and no muscle aches or other symptoms. A chest computed tomography scan (CT) (Figure 1Ba1,a2) revealed slight bronchopneumonia in the superior lobe of the right lung. On January 30, a nasopharyngeal swab tested positive for SARS-Cov-2. Laboratory examinations showed abnormally increased lymphocytes (26.9 × 10^9^/L, range, 1.1–3.2) with normal counts of hemoglobin and platelets, elevated D-dimer (3.23 mg/L, range, < 0.5), and decreased immunoglobulin A (0.75 U/L; range,0.82–4.53). A blood smear showed a large number of mature lymphocytes (Figure 1C). Immunophenotypic analysis showed that a single B lymphocyte clone accounted for 73.3% in non-erythroid cells; these cells were CD19^dim^, CD5+, CD22+, CD20+, CD23+, CD200+, and FMC7+, while kappa light chain was restrictively expressed, and CD10, CD103, and sIgM were not expressed (Figure 1D). Multiple areas of ground-glass opacities under the pleura of both lungs were observed on chest CT on the admission day (Figure 1Bb1–b3). She was diagnosed with COVID-19 and CLL. Figure 1E shows the symptoms, the results of the virus RT-PCR tests, and the treatments used for I-2, I-1, and II-3 during a follow-up period of 69 days.

On admission day 3, I-2 complained of shortness of breath, and arterial blood gas analysis revealed hypoxemia. A comprehensive analysis of the clinical manifestations and pulmonary image and a decreased neutrophil-to-lymphocyte ratio showed that the patient had developed severe pneumonia. She received a modified combination regimen composed of 800/200 mg/day lopinavir/ritonavir (LPV/r), 1,000 WU/day interferon α-2b via aerosol inhalation, 1.6 mg/day subcutaneous thymosin, 0.6 g/day arbidol (tablets), and 250 mg/kg/day intravenous immunoglobulin (IVIG) for 5 days. Chest CT revealed focal consolidation accompanied by fibrosis in the patient on day 8 (Figure 1Bc1,c2). The patient suffered secondary pulmonary bacterial infection and received anti-infective treatment on day 10. Beginning on day 13, no fever, headache, dizziness, nausea, vomiting, or other complications affecting the respiratory system were observed. Re-examination with chest CT on day 18 revealed the absorption of pneumonia (Figure 1Bd1,d2). Respiratory, serum, and stool specimens were negative for SARS-Cov-2 for 2 days according to the results of repeated RT-PCR tests. The patient recovered and was discharged on February 19.

Chest CT revealed obvious improvement of the double lung infection in the patient on day 26 (Figure 1Be1,e2). However, on day 35, a respiratory specimen was positive for SARS-Cov-2, and the patient was readmitted to the hospital for isolation, though she had no complaints. Chest CT revealed new ground-glass shadows in the patient (Figure 1Bf1,f2). Serological results revealed both 2019-nCoV-specific IgM and IgG antibodies in patient I-2; however, only specific IgG antibodies were detected in her husband's plasma. She received 1,000 WU/day interferon α-2b, 0.6 g/day arbidol (tablets), and 250 mg/kg/day IVIG for 3 days to modulate the immune response. Chest CT showed obvious absorption of the infection in the patient on day 47 (Figure 1Bg1,g2). On day 50, respiratory, stool, and serum specimens showed two successive negative results. The patient was discharged on day 52. IVIG was intravenously injected once a week after discharge. However, the patient tested positive for the virus for the third time on day 64, and she was the third admitted to the hospital for isolation. Interferon α-2b via aerosol inhalation, arbidol (tablets), and IVIG were applied on April 2. On day 67, respiratory, serum, and stool specimens were all negative for SARS-Cov-2 for 2 days once again. She was discharged again on day 69.

**Figure 2:**
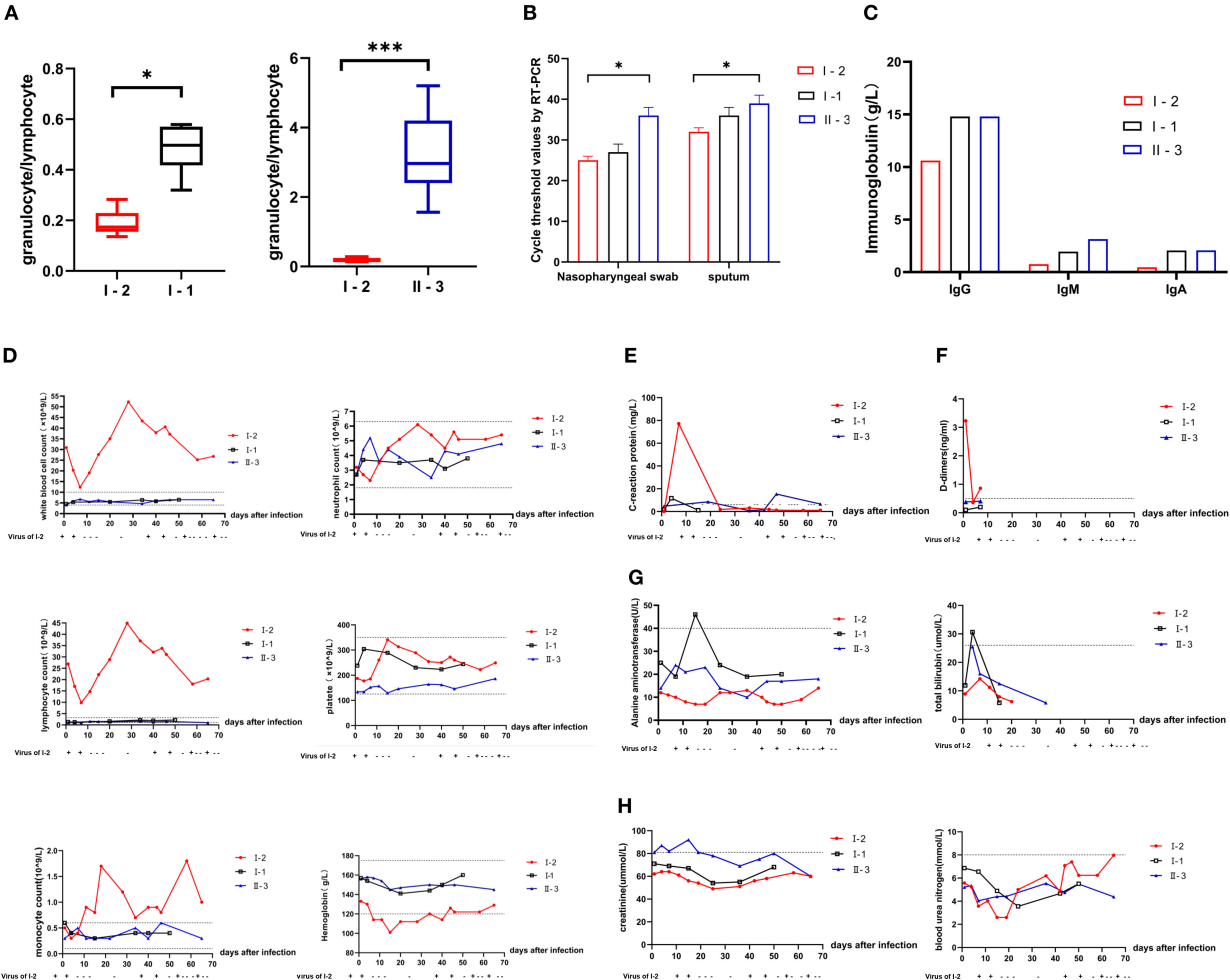
Comparison of laboratory results of patients from a household cluster with COVID-19 during 69 days of follow-up. **(A)** Trends of the neutrophil-to-lymphocyte ratio (NLR) during follow-up for 69 days. **(B)** Ct values for detection of novel coronavirus nucleic acid. **(C)** IgG, IgM, and IgA levels of I-2, I-1, and II-3 at initial diagnosis. Changes in **(D)** routine blood examination values, **(E)** C-reactive protein (CRP), **(F)** D-dimers, **(G)** liver function including galanine transaminase and total bilirubin, **(H)** renal function including creatinine and blood urea nitrogen in patients I-2, I-1, and II-3 during the 69-day follow-up. *p = 0.01–0.05 and ***p < 0.001.

**Figure 3:**
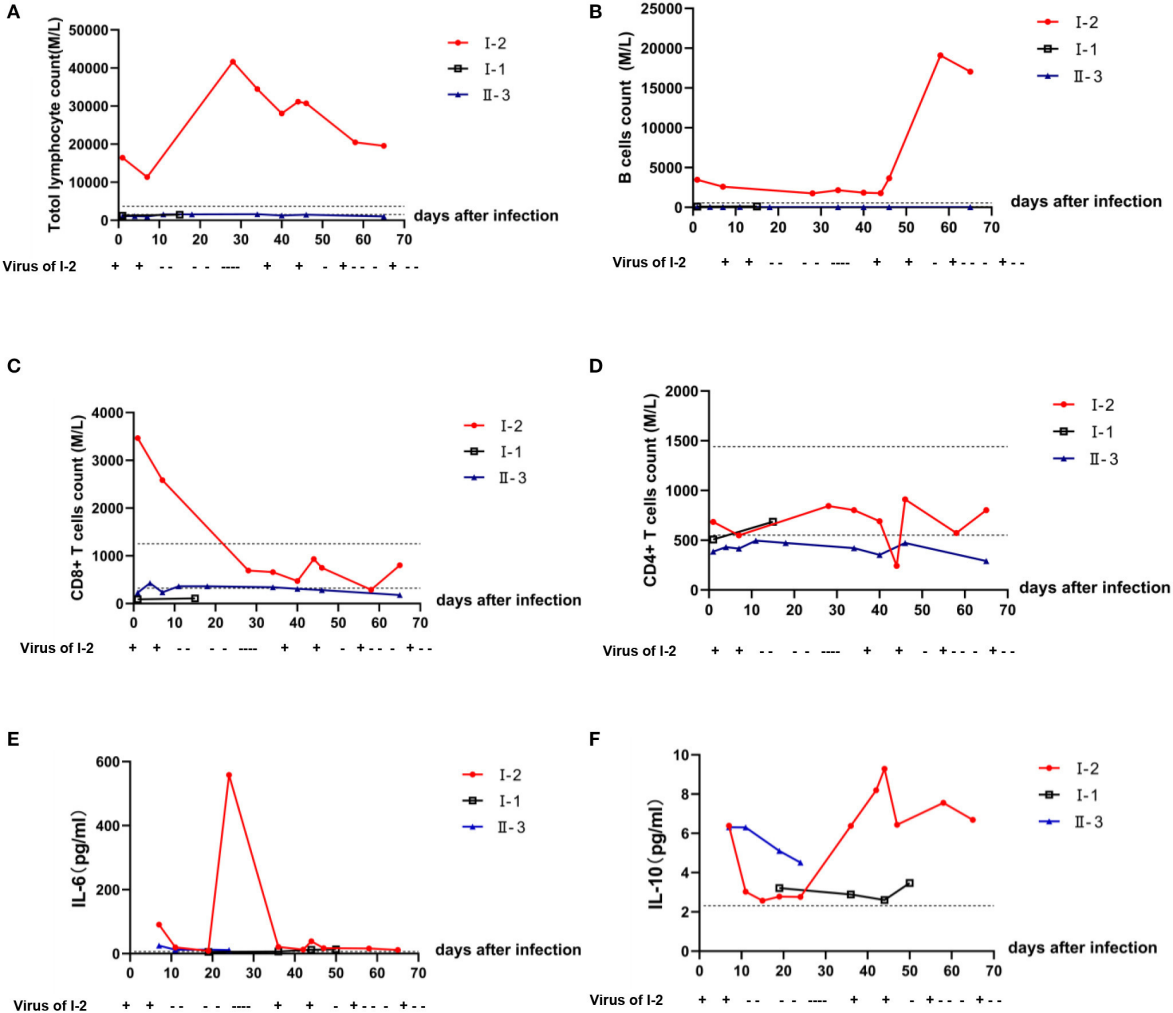
Dynamic trends in the lymphocyte subsets and cytokines in peripheral blood among patients within a household cluster with COVID-19 during 69 days of follow-up. Trends of **(A)** lymphocyte subsets; **(B)** B lymphocytes; **(C)** CD8+ T lymphocytes; **(D)** CD4+ T lymphocytes. **(E)** Level of IL-6 in peripheral blood as detected by flow cytometry; **(F)** level of Il-10 in peripheral blood as detected by flow cytometry.

Compared with that of I-1 and II-3, the neutrophil-to-lymphocyte ratio (NLR) of I-2 was significantly lower (*P* < 0.05) (Figure 2A, *t*-test, using SPSS software version 22). According to the Ct values determined with the SARS-Cov-2 virus test, I-2 had the lowest Ct value among the three patients regardless of whether the specimen was from the initial nasopharyngeal swab or initial sputum (Figure 2B). Decreased Ct values indicate increased viral load. At diagnosis, the levels of immunoglobulin (Ig) G, IgA, and IgM for I-2 were among the lowest of those of the family members (Figure 2C). Routine blood examination was performed, and C-reaction protein, D-dimer, liver function, and renal function were monitored. The white blood cell (WBC) and lymphocyte counts for I-2 were evidently high, and they dropped significantly after every recurrent novel coronavirus infection (Figure 2D). The expressions of CRP and D-dimer were significantly elevated during the process of treatment (Figures 2E,F). However, no remarkable changes were observed in hepatorenal function in I-1, II-3, and I-2 (Figures 2G,H). The dynamic trends of CD4+ and CD8+ lymphocytes, B lymphocytes, and cytokines IL-6 and IL-10 in patients I-2, I-1, and II-3 within 69 days of follow-up are shown in Figure 3. The lymphocytes were detected with flow cytometry assays, and the cytokines were measured in serum. At first, the numbers of CD4+ and CD8+ lymphocytes of I-2 were higher than those of II-3 and I-1, while the CD4+/CD8+ ratio of I-2 was lower than that of the other two subjects (Figures 3C,D). Significant increases in IL-6 levels were observed in I-2 before the first recurrence (Figure 3E), and the IL-10 levels in I-2 were high before the second recurrence (Figure 3F).

## Discussion

There are limited data available on COVID-19 patients with hematological malignancies over more than 60 days of follow-up. Our patient met the diagnostic IWCLL criteria. Monoclonal cells in CLL affect the phenotype and function of a variety of innate and adaptive immune cells, leading to an infection-prone environment and reduced immune surveillance (4, 13, 14). Studies have found humoral immune failure in CLL, which may be associated with hypogammaglobulinemia and IgG subclass deficiency (3, 15–17), as CLL cells impair the immune system (18). Several lines of evidence have indirectly indicated that antigenic stimulation through the B-cell receptor (BCR) supports CLL development (19), and patients with CLL should avoid using drugs that impair B-cell function during SARS-CoV-2 infection.

Furthermore, there is increased susceptibility to viral infections due to T-cell dysfunction in CLL patients (17). By monitoring and comparing the patient's laboratory findings with those of the other family members, we found significant differences between the immunosuppressed patient and immune-competent patients, including the counts of blood cells and of the CD4+ and CD8+ lymphocyte subpopulations and B cells in peripheral blood and the levels of cytokines after 2 months of infection. Within 10 days after infection, the WBC of I-2 decreased significantly with decreases in neutrophils and lymphocytes to a certain extent, while I-1 and II-3 had normal immune function, increased neutrophils, and a slight decrease in lymphocytes. At first, the numbers of CD4+ and CD8+ lymphocytes of I-2 were higher than those of II-3 and I-1, while the CD4+/CD8+ ratio of I-2 was lower than that of the latter two subjects. During the follow-up, a significant decrease in CD8+ lymphocytes was observed in I-2. However, minor changes in lymphocytes, including CD8+ T cells, were observed in II-3 and I-1. Interestingly, significant increases in IL-6 levels were observed in I-2 before the first recurrence, and IL-10 levels were high before the second, suggesting that high IL-6 or IL-10 levels may also predict the recurrence of SARS-Cov-2.

Multiple scattered ground-glass shadows in the lung, a high viral load, and the decreased NLR provided clues that I-2 had developed severe pneumonia. A study has shown that monitoring the NLR is helpful in the early screening of critical illness and the diagnosis and treatment of COVID-19 (20). The condition of patients with CLL should be monitored according to the NLR rather than the absolute number of lymphocytes. Liu et al. (20) reported that the degree of lymphopenia and the severity of proinflammatory cytokine storms are increased in severe COVID-19 patients compared with those in patients with mild cases. Severe patients showed significant and sustained decreases in lymphocyte and T-cell counts and increases in IL-6, IL-10, IL-2, and IFN-γ levels, while mild cases exhibited increased neutrophil counts (12, 21). The clinical characteristics in our case were consistent with the features of severe cases.

Owing to humoral immune dysfunction, complete antibodies are improperly formed. Based on the inhibition of innate immune surveillance and the dysfunction of acquired immunity, clearance of the virus showed a delay in I-2 in comparison with I-1 and II-3. Complete antibodies were not formed, and weakened cell-killing effects prevented the virus from being completely cleared and led to repeated recurrence. Recurrent positivity for SARS-Cov-2 will likely occur in I-2 for a longer period of time. High-dose IVIG combined with antiviral therapy seems to be an effective treatment to alter the inflammatory response and prevent the development of severe pneumonia.

## Conclusion

In CLL patients, a neoplastic antigen-specific B-cell clone proliferates and outgrows other B cells, leading to immune deficiency. Considering the low humoral immune response and ineffective clearance of SARS-Cov-2 in CLL patients, the follow-up and home quarantine period should be extended for such patients. The treatment of CLL may lead to further weakening of humoral immunity. Therefore, we need further studies to clarify whether CLL therapy should be suspended or continued during COVID infection. For those patients who are prone to progression into a severe stage, administering humoral immunity therapies can help to prevent disease progression and quickly meet the cure criteria.

## Data Availability Statement

The raw data supporting the conclusions of this article will be made available by the authors, without undue reservation.

## Ethics Statement

The studies involving human participants were reviewed and approved by the Ethics Committee of the Fourth Affiliated Hospital of Zhejiang University. The patients/participants provided their written informed consent to participate in this study. Written informed consent was obtained from the individual(s) for the publication of any potentially identifiable images or data included in this article, and for the publication of this case report.

## Author Contributions

XY, XX, BL, and JH: performed the research and wrote the manuscript. WZ and YL: performed the experiments and analyzed the data. JW and XH: assisted with laboratory experiments and provided technical advice. JinJ and DC: contributed to patient samples and assisted with data collection. JieJ and JH: designed the study and assisted in manuscript preparation. All authors approved the manuscript for submission.

## Conflict of Interest

The authors declare that the research was conducted in the absence of any commercial or financial relationships that could be construed as a potential conflict of interest.
